# Optimized Grasshopper Optimisation Algorithm enabled DETR (DEtection TRansformer) model for skin disease classification

**DOI:** 10.1371/journal.pone.0323920

**Published:** 2025-05-29

**Authors:** Shakti Kundu, Yogesh Kumar Sharma, Khan Vajid Nabilal, Gopalsamy Venkatesan Samkumar, Sultan Mesfer Aldossary, Shanu Kuttan Rakesh, Nasratullah Nuristani, Arshad Hashmi

**Affiliations:** 1 School of Engineering and Technology, Computer Science Engineering, BML Munjal University Gurugram, Gurugram, Haryana, India; 2 Department of Computer Science & Engineering, Koneru Lakshmaiah Education Foundation, Guntur, Andhra Pradesh, India; 3 Genba Sopanrao Moze College of Engineering, Pune, Maharashtra, India; 4 Computer Engineering Department, College of Engineering, Prince Sattam Bin Abdulaziz University, Wadi Aldawaser, Saudi Arabia; 5 Department of Computer Science and Engineering, Chouksey Engineering College, Bilaspur, Chhattisgarh, India; 6 Department of Spectrum Management, Afghanistan Telecommunication Regulatory Authority, Kabul, Afghanistan; 7 Department of Information Systems, Faculty of Computing and Information Technology in Rabigh (FCITR), King Abdulaziz University, Jeddah, Saudi Arabia; Prince Mohammad Bin Fahd University, SAUDI ARABIA

## Abstract

Skin disease classification is a choir cognate for early diagnosis and therapy. The novelty of this study lies in integrating the Grasshopper Optimisation Algorithm (GOA) with a DETR (DEtection TRansformer) model which is developed for the classification of skin disease. Hyperparameter tuning using GOA optimizes the critical parameters of the proposed model to improve classification accuracy. After extensive testing on a large dataset of skin disease photos, the optimised DETR model returned an accuracy of at least 99.26%. The superiority of the DETR improved using GOA compared to standard ones indicates its potential to be used for automatically diagnosing skin diseases. Findings demonstrate that the proposed method contributes to enhancing diagnostic accuracy and creates a basis for improving transformer-based medical image analysis.

## 1. Introduction

Skin diseases are one of the most common health problems globally, across all ages. As a result of the many different forms from simple infections to serious skin conditions like skin cancer, their prediction is now an important area of research. Holistic health: Early prediction improves patient care and prognosis with lower complication risk and better chronic skin condition management. Due to the growth of machine learning (ML), the prediction of skin disease is moving more automatically which can accompany the rapid identification and accurate skin disease compared to the early process of diagnosis that use conventional methods. In the past, diagnosing skin disease requires only dermatologists to visually check the area and later on, confirm with biopsies or lab tests. The problem is that this method is not only time-consuming but also can lead to misdiagnoses as dermatologists can vary in their experience and expertise [1–3]. AI and ML models, specifically deep learning-based models, have emerged as a great solution for dermatology image analysis and the detection of diseases with high accuracy. Large datasets of labeled skin images can be used to train algorithms that learn to identify patterns indicative of specific conditions such as psoriasis, eczema, acne, or melanoma. Deep learning, especially CNNs, has proved to be an excellent way of inferring skin disease. CNNs automatically extract features from images, which may not be visible to the human eye, they are optimized to run with images [4]. CNNs can identify these small differences in texture, colour or shape in skin lesions that are suggestive of melanoma malignancy and psoriasis chronicity as an example. These models have been shown to classify skin diseases with accuracy comparable to, and in some cases superior to, that of experienced dermatologists in a controlled study. One of the primary aspects of prediction models for skin diseases is that all of them depend on quality data, that too across a diverse population [5] The appearance of skin diseases can vary according to skin, age, sex, and geographical position. The data used in training, in most of the cases, tend to be biased towards light skin which are resulting in misdiagnosis of the people of colour. This creates an ethical problem, as it indicates the necessity to have diversity and class in these datasets to have an AI for the good of every patient in the world, whatever their ethnic group or origin [6].

Visual analysis is one part of the puzzle and one factor alone is not sufficient to determine the cause of skin disease and, in addition to genetic predisposition, environmental triggers and lifestyle choices contribute to its development. Psoriasis and eczema have a genetic predisposition and the risk of skin cancer rises after too long an exposure to UV rays. Many predictive models are currently being developed that analyze dermatological images along with genetic, environmental, and personal data to give a more holistic prediction of skin diseases [7]. By providing personalized treatment plans and early interventions, these multimodal models could radically shift the paradigm of personalized dermatology. Apart from the technical challenges of developing precise prediction models, there are also practical and ethical concerns [8–10]. Combining genetic data introduces serious privacy issues due to the sensitive nature of the patient data being collected. Furthermore, AI-based systems should not replace dermatologists, but rather, tools to assist them. Expert oversight of AI predictions is needed for the interpretation of results because a misdiagnosis means an inappropriate treatment, worsening the illness [11].

### 1.1. Problem formulation

There are other challenges faced specifically by skin disease prediction models such as they demand real-time application too. Such a model needs to be fast and reliable to predict skin diseases in clinical settings. Novel mobile health (mHealth) technologies are in development that allow patients to “take a picture of their skin and get an answer” within minutes. Such tools could make it easier to access dermatology services, especially in less developed parts of the world. Nevertheless, mhealth apps should be accurate enough to guide users to follow up with health professionals, when indicated. Explainability in AI models is an active research area to improve the precision and accuracy of skin disease prediction. However, medical dermatologists are reluctant to believe black-box algorithms which reveal very little or nothing at all about how to get to a diagnosis (Haegeman et al., 2020). eXplainable Artificial Intelligence (XAI) also aims to clarify how these models work, enabling dermatologists to not just utilize predictions, but understand the reason behind those predictions to help them with their decision-making process. Researchers hope such increased interpretability of AI tools can allow for enhanced human-machine partnership in the clinic. AI and ML technologies have great potential for the prediction of skin diseases [12]. These innovations, from the use of CNNs for image studies to more holistic predictions through the incorporation of multimodal data, can help with earlier detection, improved chronic condition management, and more personalized treatment plans [13–15]. However, the treatment of patient data by these technologies needs to be fair, transparent and ethical if they are to be implemented and widely adopted. For this reason, the ultimate intention is not to replace dermatologists but to offer them powerful tools that enhance their diagnostic skills and improve patient outcomes across the globe [16–18].

Our contributions can be summarized as follows:

- A modified DETR model is proposed to classify various skin disease.
- Efficient hyperparameter tuning mechanism based on Grasshopper Optimisation Algorithm tunes the DETR model to classify various skin disease.
- This proposed algorithm provides a new idea for classify various skin disease.

This paper is designed with aiming of classifying various skin disease. Section 2 defines the various existing works carried out in this problem domain. Section 3 explains the datasets used for experimental purposes. In this paper, the skin disease images dataset is being taken. Section 4 demonstrated the classification of various skin disease through a modified DETR model. Next, section 5 illustrates the result, followed by the paper’s conclusion in section 6.

## 2. Related work

Cula et al. (2004) developed bidirectional skin imaging, which captured more appearance properties than normal imaging. The angle of incident illumination and observation considerably affected skin structure. The Rutgers Skin Texture Database (clinical component) used bidirectional imaging methods. This was dermatology’s first picture database. Public skin photos of numerous illnesses under controlled illumination and viewing orientations were made available for study and teaching. We automated skin texture classification using computational surface modeling with this database. Classification trials showed the utility of modeling and measuring methods [10].

Using cutting-edge image processing, Khan et al. (2019) presented an intelligent approach to differentiate melanoma from nevus. First, the Gaussian filter removed noise from the skin lesion images, and then better K-mean clustering segmented them. Extracting texture and color features from the lesion created a unique hybrid superfeature vector. SVM classified skin cancer as melanoma or nevus. We aimed to evaluate the segmentation methodology, extract the best features, and compare the classification results to other methods in the literature. The suggested technique was tested on 397 skin cancer photos from the DERMIS collection, including 146 melanoma and 251 nevus conditions. Our technique yielded a promising 96% accuracy [25].

Kawahara et al. (2019) trained a multitask deep convolutional neural network on clinical and dermoscopic pictures and patient metadata to classify the 7-point melanoma checklist and identify skin lesions. Multitask loss functions trained the neural network for multiple input modalities, making it resilient to missing data at inference time. The final models classified the 7-point checklist, diagnosed skin diseases, built multimodal feature vectors for image retrieval, and identified clinically discriminant regions [23].

Zhang et al. (2019) created an ARL-CNN dermoscopy skin lesion classification model with ARL blocks, a global average pooling layer, and a classification layer. Residual and creative attention learning improved discrimination in each ARL block. The proposed attention learning method built low-layer attention maps from high-layer feature maps using DCNNs’ self-attention instead of learnable layers. The ARL-CNN model was tested at ISICskin 2017. The results suggested that the ARL-CNN model could adapt to skin lesion discriminators for advanced classification [58].

Adegun & Viriri (2020) segmented and classified skin lesions to detect skin cancer automatically. From difficult and inhomogeneous skin lesion data, an encoder-decoder Fully Convolutional Network (FCN) learned coarse appearance and lesion boundary properties. The typical FCN used long skip connections for residual learning and successful training, while their FCN featured sub-networks connected by skip routes with long skip and short-cut connections. The Conditional Random Field (CRF) module refined contours and localized lesion boundaries using linear Gaussian kernels for paired edge potentials. The second stage introduced an FCN-based DenseNet framework with dense blocks connected by concatenation and transition layers. Hyper-parameters were optimized for network simplicity and processing efficiency. Feature reuse required minimal data and settings. On the publicly accessible HAM10000 dataset of over 10,000 images categorized by seven illnesses, the model achieved 98% accuracy, 98.5% recall, and a 99% AUC score [1].

Ahmad et al. (2020) used a triplet loss function to fine-tune ResNet152 and InceptionResNet-V2 for their unique framework. Images were placed in 3D using deep convolutional neural network (CNN) ResNet152 and InceptionResNet-V2 models. Second, they used the triplet loss function to determine the L-2 distance between relevant images in Euclidean space to learn how to distinguish skin conditions in photos. After that, input photos were sorted by L-2 distances. Wuhan Hospital provided face skin disease images for the framework. Experiments revealed that the proposed framework was more accurate than earlier skin disease task frameworks [3].

Pham et al. (2020) proposed a hybrid approach to skin disease classification with class imbalance. This approach relied on algorithm-level loss function design, data-level balanced mini-batch logic, and real-time image augmentation. The largest skin cancer dataset, the training dataset, had 24,530 dermoscopy photos of seven skin diseases. Six techniques were evaluated using 2,453 photos. The highest accuracy, mean recall, and standard deviation model was EfficientNetB4-CLF (89.99%). They decreased recall standard deviations by 4.24% (±11.84% to ±7.60) and outperformed previous methods by 4.65% (86.13% vs. 81.48%). Thus, their hybrid technique trained Deep CNN on the imbalanced skin disease dataset, incorporating data-level balanced mini-batch logic that augmented photos in real-time and algorithm-level newly-built loss functions to benefit under-represented classes [33].

Using Back et al.‘s (2021) knowledge distillation from ensemble via curriculum training (KDE-CT), students benefited from better instructors. Compared to the HZ diagnostic skin disease dataset, 75 corruption types existed. Thirteen DNNs were tested on undamaged and degraded pictures. Trial results showed that KDE-CT improved corruption robustness more than other approaches. Their MobileNetV3-Small outperformed the DNN ensemble in mobile skin lesion analysis with 549 times fewer multiply-and-accumulate operations (93.5% accuracy, 67.6 mean corruption error) [6].

Senan and Jadhav (2021) developed an automatic ABCD skin cancer diagnosis algorithm. PH2, which comprised Atypical, Melanoma, and Common Nevi, was analyzed using the indicated technique. The proposed solution used Gaussian filters to remove superfluous pixels from photos during pre-processing. The contouring technique recovered RoI from dermoscopy pictures. Morphology improved lesions. The second step retrieved relevant characteristics using ABCD rules. The Total Dermoscopy Score distinguished benign from malignant characteristics. The proposed system’s performance was measured using the above metrics [23].

Khan et al. (2021) recommended HDCT-based saliency segmentation using a 16-layered convolutional neural network on binary pictures. RGB lesion images were created from binary photos using maximal mutual information. The classification module retrained DenseNet201 from segmented lesion images using transfer learning. T-SNE reduced two fully connected layer characteristics. A fused multi-class ELM classifier used features from MCCA. Classification used HAM10000, the toughest dataset, while segmentation used ISBI2016, ISIC2017, PH2, and ISBI2018. Experimental results favored their framework over current methods [24].

Ahammed et al. (2022) deblurred images using Gaussian filtering and extracted digital hair with Black-Hat transformation and inpainting. Automatic Grabcut segmentation isolated troublesome lesions. Statistics and GLCM extracted skin photo input patterns. The ISIC 2019 challenge and HAM10000 validated models. Decision Tree (DT), Support Vector Machine (SVM), and K-Nearest Neighbor (KNN) classifiers classified skin images as melanoma (MEL), melanocytic nevus (NV), basal cell carcinoma (BCC), actinic keratosis (AK), benign keratosis (BKL), dermatofibroma (DF), vascular lesion (VASC), and Squam SVM. Their work was compared to newer methods [2].

Yao et al. (2022) described a single-model-based skin lesion classification method for limited and unbalanced datasets. Many DCNNs were trained on tiny, imbalanced datasets to demonstrate that moderately complex models outperformed larger ones. DropOut and DropBlock decreased overfitting, and Modified RandAugment corrected small dataset sample under-representation. Lastly, end-to-end CLS and a novel Multi-Weighted New Loss (MWNL) function addressed unequal sample sizes, classification difficulty, and anomalous sample training effects [56].

Lee et al. (2023) developed a Multi-modal smartphone imaging system including RGB and fluorescence images to diagnose skin diseases using an auxiliary deep learning network—fluorescence-aided amplification network (FAA-Net). They incorporated an attention-based module into FAA-Net that self-localized skin diseases and identified potential disease locations. Their multi-modal system was used in a hospital clinical trial to evaluate FAA-Net and compare its performance to existing state-of-the-art skin disease diagnosis models. Their model outperformed other sophisticated models in skin disease classification by 8.61% and 9.83% in mean accuracy and area under the curve [28].

Wang et al. (2023) proposed autoSMIM, a self-supervised superpixel-based masked image modeling method for skin lesion segmentation. They examined implicit features in several unlabelled dermoscopic pictures. Their model was tested on three skin lesion segmentation datasets—ISIC 2016, 2017, and 2018. Their autoSMIM outperformed state-of-the-art approaches [52].

Su et al. (2024) proposed a two-stage GAN-based Self-Transfer GAN to create unique skin lesion images for imbalanced datasets. Their STGAN-based framework successfully classified with high accuracy [46].

The skin lesion classification literature in Table 1 has numerous major research gaps that must be addressed to improve diagnostic performance. Some research, as Khan et al. (2019) with the DERMIS dataset, use small datasets that may overfit and not generalise effectively to bigger populations. Many models, like Kawahara et al.‘s (2019) multitask deep convolutional neural network, struggle with label differentiation, underscoring the need for more advanced classification methods. Zhang et al. (2019) lack unsupervised attention learning, indicating a need for the use of sophisticated machine learning methods to improve model interpretability and robustness. This pattern continues across techniques, as Adegun & Viriri (2020) found constraints in completeness and exactness and numerous models had low performance metrics across datasets. Existing models have systemic computing efficiency and scalability issues. Pham et al.’s (2020) EfficientNetB4-CLF model had 89.97% accuracy but needed to redefine the loss function to improve model training. According to Back et al. (2021), Lee et al. (2023), and Su et al. (2024), these models are impractical in real-world situations due to high computing costs. Class imbalance complicates diagnostic system development, as Yao et al. (2022) noted. For automated skin lesion categorisation to advance, larger, more diversified datasets, new algorithmic techniques, and computational efficiency must be integrated.

**Table 1:** Summary of related work.

S. No.	Authors	Models	Dataset	Accuracy	Limitations
1	Khan et al. (2019)	K-mean clustering	DERMIS dataset	96%	Small Dataset
2	Kawahara et al. (2019)	Multitask deep convolutional neural network	1011 lesion cases	89.6%	Unable to distinguish among the labels
3	Zhang et al. (2019)	Deep convolutional neural networks (DCNNs)	ISICskin 2017 dataset	89.8%	Missing Unsupervised attention learning
4	Adegun & Viriri (2020)	Fully Convolutional Network (FCN)	HAM10000 dataset	98%	Lower measure of completeness and exactness
5	Ahmad et al. (2020)	ResNet152 and InceptionResNet-V2 models	6144 images from Wuhan Union Hospital	87.42%	Low performance
6	(Pham et al., 2020b)	EfficientNetB4-CLF model	24,530 dermoscopic images	89.97%	The loss function needs to be redefined
7	Back et al. (2021)	Deep neural network (DNN)	SD-HZ dataset	93.5%	Heavy computational cost
8	(Senan & Jadhav, 2021a)	ABCD rules	PH2 dataset	84%	Low performance
9	(Khan et al., 2021a)	DenseNet201 model	ISBI2016, ISIC2017, PH2, and ISBI2018 dataset	96.6%	High redundancy
10	(Yao et al., 2022)	Deep convolutional neural network (DCNN)	ISIC 2018 dataset	87.5%	Class imbalance issue
11	(Lee et al., 2023a)	Fluorescence-aided amplifying network (FAA-Net)	ISIC2020 dataset	81.88%	Heavy computational cost
12	Su et al. (2024)	Generative Adversarial Networks (GANs)	HAM10000 dataset	98.23%	Heavy computational cost

## 3. Materials and methods

### 3.1. Dataset

**Fig 1:**
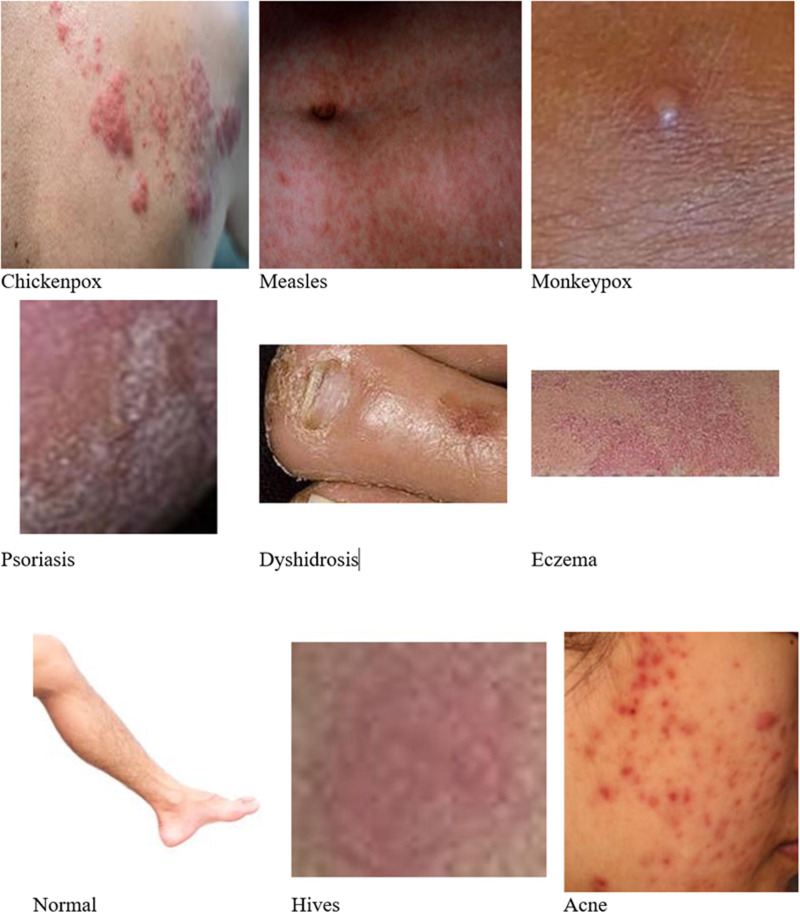
Various skin diseases.

The Skin disease dataset contains 1601 images across nine classes of skin ailments; Acne, Chickenpox, Dermatitis Eczema Hives Measles Monkeypox Normal Psoriasis. This dataset consists of nine classes of skin conditions with varying numbers of images per category. Acne has the highest representation with 456 images, followed by normal skin (292), eczema (300), and dermatitis (285), indicating a strong focus on common skin issues. Hives (91), monkeypox (78), chickenpox (58), and measles (31) have fewer images, likely due to their relative rarity or difficulty in data collection. Psoriasis has the lowest count at just 10 images. The dataset is split into 3 parts; Training set (70% of the data, i.e., 1,124 images), Validation Set (20% of the data, i.e., 316) and Test Set (10% of the paintings, i.e.,161). Sample sizes of each subset and from all classes are chosen such that the model can learn heterogeneous characteristics of a skin condition as opposed to being merely focussed on some traits only, likewise ensuring robust performance over different conditions shown in Fig 1.

**Fig 2:**
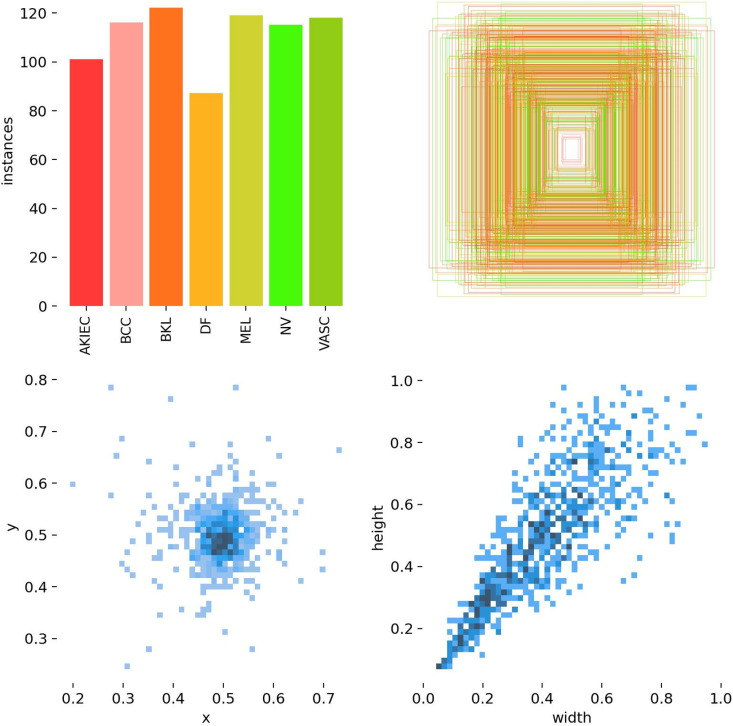
Skin disease size distributions.

Before the authors could train the model, some preprocessing was needed. Every image was auto-rotated to right any orientation differences and resized at a uniform dimension of 640x640 pixels to keep the dataset standard onstage shown in Fig 2.

**Fig 3:**
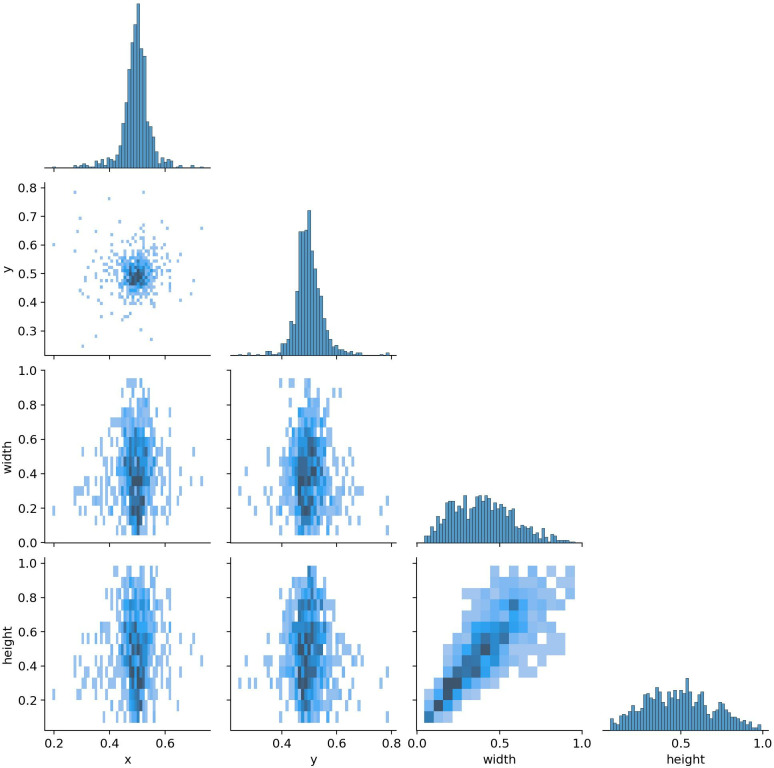
Skin diseases instance distributions.

This resizing was done by stretching to match the input shape for models. In this manner, the authors avoided a significant source of variation that could have been introduced by image dimensions and orientation. However, no other augmentations—such as rotations, flips, or color adjustments—were applied, as shown in Fig 3.

While some classes are underrepresented in the dataset in comparison to others, class imbalance becomes an issue while training object detection models. This problem is not explicitly provided as a customizable option, although YOLOv8 has internal ways to alleviate it during training. A key component of YOLOv8’s approach to addressing class imbalance is Focal Loss, which automatically prioritizes more difficult and less common instances over simpler and more common ones. When working with datasets that are unbalanced, this can be useful for redistributing the emphasis of learning. To further aid in providing a fairer picture of diverse classes, YOLOv8 employs data augmentation procedures during training to artificially enhance underrepresented classes more frequently. A larger effective sample size and more variability for minority classes in this dataset may be achieved by augmentations including random scaling, cropping, and flipping.

### 3.2. Methods

Object detection is a computer vision technique for identifying and locating objects within images or video frames. Object detection combines two primary tasks:

- Object Classification: Identifying what objects are present in the image.
- Object Localization: Determining where each object is located within the image, typically by drawing bounding boxes around them.

By detecting and identifying objects in images, object detection helps make sense of visual information in various fields, such as automatic tagging, automobile safety, retail, security, and more. DETR is a deep learning model for object detection that leverages transformers to predict bounding boxes and class labels for an image. Transformers are neural networks consisting of an encoder-decoder architecture with self-attention mechanisms [15–18]. An essential part of the DETR design, the’self_attention_layer’ models long-range relationships in the input data to help with skin disease feature identification and categorization. To help the model zero in on the important parts of the input picture and ignore the rest, this module calculates attention scores by comparing the query, key, and value representations of the input features. With transformers, DETR can capture global context and relationships between objects in an image, leading to more accurate detections. Traditional object detection approaches like YOLO (You Only Look Once) and R-CNN (Region Convolutional Neural Network) generate many region proposals or anchor boxes and filter them using Non-Maximum Suppression (NMS). This approach makes the architecture complex and training inefficient [19–21].

**Fig 4:**
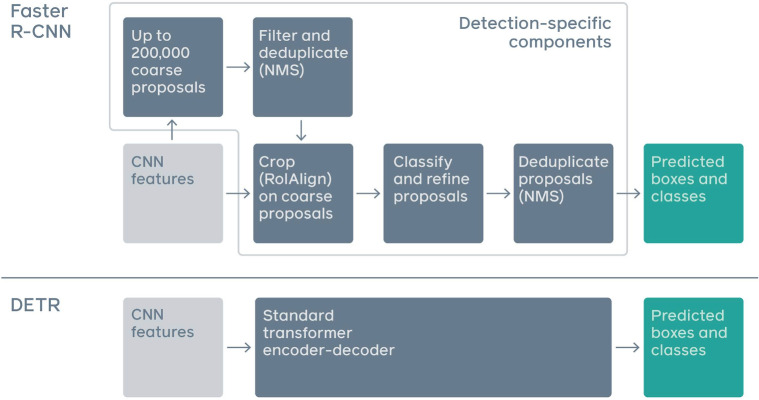
Difference between pipelines of Faster R-CNN and DETR.

Fig 4 depicts that DETR makes object detection a direct set prediction problem. Its goal is to consider all the objects in the image as a set and predict their bounding boxes and classes in a single pass. This new approach improves the detection accuracy, especially when many objects are close to each other.

**Fig 5:**
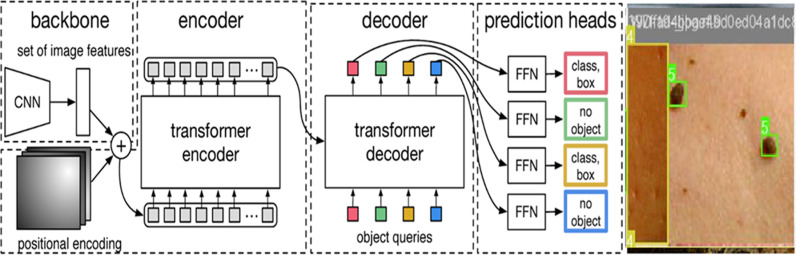
DETR architecture.

The main components of DETR architecture are the Convolutional Neural Network (CNN) backbone, the transformer encoder and decoder, and the prediction heads. The DETR architecture is shown in the Fig 5 below:

Fig 5 demonstrates the DETR architecture, which consists of the following components:

- CNN Backbone: The image is first passed through a CNN backbone which outputs feature representations of the image at a high level. Some examples of popular CNN backbones are Visual Geometry Group (VGG) and ResNet. These features contain spatial information about the objects in the image, which is then passed as input to the transformer encoder [22–24].
- Transformer Encoder: The transformer encoder encodes these features into a sequence of feature vectors. As the encoder contains multi-headed self-attention blocks, it allows for capturing contextual information through long-range dependencies between different parts of an image. At this stage, another crucial element of the pipeline is the addition of positional encodings to CNN’s output. As transformers do not inherently have spatial understanding, these positional encodings inform them about the relative positions of the objects in the image [25].
- Transformer Decoder: The transformer decoder learns relationships between the CNN-encoded features and the learnable object queries. Typically, we require queries, keys, and values to calculate self-attention in NLP [26]. Therefore, in computer vision, DETR introduces the concept of object queries, which refer to the learnable representations of the objects the model needs to predict. The number of object queries is predetermined and remains fixed. The keys denote the spatial locations in the image, while the values contain information about the features [27–29].
- Prediction Heads: The prediction heads output the bounding boxes and classes of the detected objects. They consist of feed-forward network heads that either predict the bounding box and class for the detected objects or a ‘no-object’ class for no detections. Furthermore, DETR employs the technique of bipartite matching to ensure that the predicted bounding boxes are associated with the ground truth objects. This method also helps refine the model training.

#### Training loss.

One of the main components of the training pipeline is the training loss. The training loss combines classification and regression losses as the model predicts bounding boxes and classes [30–33].

1) Set Prediction Loss: DETR uses a set prediction loss to measure the accuracy of the predicted classes of objects. We get an idea about the missing object classes by calculating the difference between the predicted and ground-truth object classes.
2) Bounding Box Loss: DETR uses a bounding box loss, which measures the disparity between the predicted and ground-truth coordinates of bounding boxes. This loss helps locate the objects precisely in the image.

DETR eliminates the need for multi-stage pipelines by performing bounding box and class prediction all in one go. It streamlines the pipeline of object detection through its end-to-end approach. As transformers use a self-attention mechanism, DETR can capture global context by looking at the positions and relations of other objects in the image, as opposed to earlier object detection methods, which predicted each object in isolation. Previous architectures, such as recurrent neural networks, made predictions sequentially, which made them slower and less efficient [34–37]. However, with the set-based approach, DETR makes the final set of predictions in parallel, which makes the training pipeline simpler and more efficient. DETR is a transformer-based model, so it requires high computational resources to train, especially when the data size is huge with high-resolution images or the backbone model is large. DETR requires mentioning a fixed number of object query counts beforehand, which can limit scenes requiring predicting a variable number of objects. Adjusting the model to handle different object counts dynamically is a challenge. While DETR simplifies the training pipeline, the inference speed can be slower than traditional methods due to the transformer architecture’s complexity, making it unsuitable for real-time use cases [38–41].

### 3.3. Proposed methodology

Hyperparameter tuning is important in the object detection tasks to achieve better results on the DETR model. Many parameters in DETR have a big impact on both transformer and convolutional backbone so it is worth to trainable hom based on training code when the authors apply them test_time for better speed/accuracy trade-off. The key hyperparameters include the learning rate, batch size, positional encodings applied (Fourier/random), transformer depth/width and number of object queries. Every parameter will be tuned according to the complexity of the dataset, object sizes and computational resources. the number of object queries (because this tells the authors allowing for how many objects the model has learned), especially important in dense detection. In this way, one can fine-tune DETR performance for a wide range of detection tasks by configuring these parameters methodically [42–45].

DETR is a combination of transformer and CNN backbone therefore learning rate is one of the most important hyperparameters for training DETR This is because a very high learning rate could result in the model converging too fast but may not be able to capture fine details whereas an excessively low learning rate would take close enough forever, causing its countless training epochs. A common method is to start with a warm-up phase where it increase the learning rate iteratively, followed by gradually decaying it using cosine annealing or step decay schedule. This enables the model to quickly learn in its initial stages of training and also helps in slowly converging towards finer weight updates which will capture complicated relationships present within the dataset [46–48].

There are the following steps of the DETR model which illustrates the main workflow from the input image to final object detection outputs.

Step 1. Begins with the input image provided to the model.

Step 2. The CNN extracts spatial features, which form a feature map.

Step 3. Feature maps are flattened to be processed in sequential form.

Step 4. Positional encoding is added to preserve spatial information.

Step 5. Encodes relationships within feature maps using self-attention.

Step 6. Initializes object queries to detect objects in the image.

Step 7. Decodes object queries about encoded features, matching objects with queries.

Step 8. The class and bounding box heads output object classes and positions.

Step 9. Final detections, including object class labels and bounding boxes, are output.

**Fig 6:**
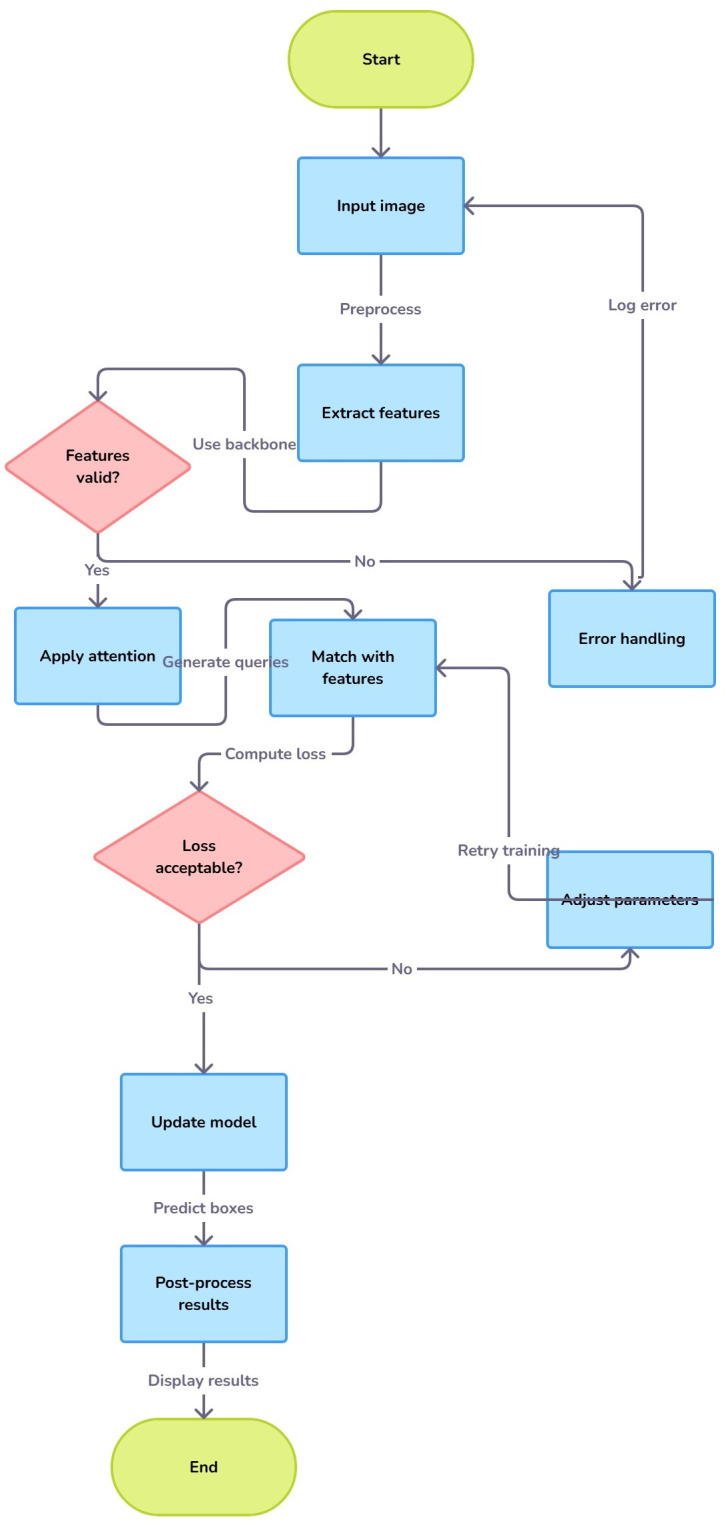
Steps of the DETR model.

These steps are shown in Fig 6. For DETR typically is recommended to use optimizers like AdamW which can deal with the very sparse updates of gradients that we get from transformers better than classic optimizers.

The pseudocode of the DETR model is as below:

# Initialize DETR components

# Backbone: CNN to extract image features

# Transformer: Encoder-Decoder model for sequence processing

# Prediction Heads: Linear layers for object class and bounding box prediction

def DETR(input_image):

# STEP 1: Extract Features using Backbone

 features = CNN_Backbone(input_image) # Shape: (batch_size, feature_dim, height, width)

# STEP 2: Flatten features and add positional encodings

 pos_encoding = generate_positional_encoding(features.shape) # Positional encoding matrix

 features = add_positional_encoding(features, pos_encoding) # Shape: (batch_size, feature_dim, seq_length)

# STEP 3: Transformer Encoder

 encoded_features = TransformerEncoder(features) # Shape: (batch_size, seq_length, embed_dim)

# STEP 4: Initialize Object Queries for Decoder

 object_queries = initialize_queries(num_queries, embed_dim) # Shape: (num_queries, embed_dim)

# STEP 5: Transformer Decoder

 decoder_output = TransformerDecoder(object_queries, encoded_features, pos_encoding)

# STEP 6: Prediction Heads

 # Class Prediction Head

 class_logits = ClassPredictionHead(decoder_output) # Shape: (num_queries, num_classes)

 # Bounding Box Prediction Head

 bbox_preds = BBoxPredictionHead(decoder_output) # Shape: (num_queries, 4), each box: (cx, cy, w, h)

 return class_logits, bbox_preds

# Function: CNN Backbone to extract image features

def CNN_Backbone(input_image):

 # Extract spatial features from the input image

 return CNN(input_image) # Shape: (batch_size, feature_dim, height, width)

# Function: Generate positional encoding matrix

def generate_positional_encoding(shape):

 # Generate a fixed or learned positional encoding based on spatial dimensions

 return positional_encoding_matrix # Shape: (feature_dim, seq_length)

# Function: Add positional encodings to flattened features

def add_positional_encoding(features, pos_encoding):

 # Add positional encoding to feature embeddings

 return features_with_pos_encoding # Shape: (batch_size, seq_length, embed_dim)

# Transformer Encoder

def TransformerEncoder(features):

 for layer in encoder_layers:

  features = self_attention_layer(layer, features)

 return features

# Transformer Decoder

def TransformerDecoder(object_queries, encoded_features, pos_encoding):

 for layer in decoder_layers:

  object_queries = cross_attention_layer(layer, object_queries, encoded_features, pos_encoding)

 return object_queries

# Prediction Heads

def ClassPredictionHead(decoder_output):

 return LinearLayer(decoder_output) # Output class scores for each query

def BBoxPredictionHead(decoder_output):

 return LinearLayer(decoder_output) # Output bounding box coordinates for each query

# Training Loop

for epoch in range(num_epochs):

 for input_image, target_boxes, target_classes in training_data:

  # Forward pass

  class_logits, bbox_preds = DETR(input_image)

  # Calculate loss

  loss = compute_loss(class_logits, bbox_preds, target_boxes, target_classes)

  # Backpropagation

  loss.backward()

  optimizer.step()

The so-called batch size affects the stability of training and convergence speed, which is another very important factor for DETR. Larger batch sizes have the effect of stabilising training as they give a better estimate of what direction and how large should this gradient be, but that does come at a cost (albeit memory). When we are able, it benefits DETR to use relatively large batch sizes; however, gradient accumulation is a good alternative when memory resources become an issue. Sometimes the authors can increase the learning rate by a small factor if they then also increase batch size, it makes training run faster while maintaining stability. Thus, finding the right tuning between batch size and learning rate is a way that can improve both speed of training as well as accuracy.

The model structure of the DETR transformer itself (the number of layers in the encoder/decoder those depths and widths) is also very critical for detection performance. The depth of a Transformer, defined by the number of encoder and decoder layers, directly affects how deeply the model processes spatial relationships, which is crucial for accurately distinguishing overlapping objects. This could be improved by adding more layers, but that also just makes it computationally harder. Similarly, the width (the number of attention heads and hidden dimensions) controls how rich those representations can be learned by our model being detailed multi-head attention. In general, an increase in heads helps the model focus on the finer details of objects, but it also overfits faster to smaller datasets. Typically these parameters are tuned to get the right balance between model capacity and dataset complexity for best performance. Having positional encoding and the number of object queries as hyper-parameters is specific to DETR. Positional encodings are important for preserving spatial information, which is especially critical in the context of object detection where objects must be localized based on their relative positions. The original DETR uses fixed positional encodings, so tests whether learned position encoding would help the model better adapt to different datasets by offering more flexibility. Another important parameter is the number of object queries: more instances that can be detected per query but significantly so computational overhead increases. This prevents a selection of harder query numbers and an easier one, determining the mean object number per image in the dataset (which forces more object matching as detection precision goes up).

Enhancing the speed of DETR is crucial for its practical application, especially in time-sensitive scenarios or with resource-constrained devices. Recent advancements such as the DETR have significantly improved the ability of its model towards object detection which learns how to predict the bounding box coordinates of the objects within an image (as opposed to complex anchor-based designs), leveraging the power captured via transformers and CNNs to do so [49–51]. However, compared to architectures like the faster R-CNN or YOLO, this special architecture has a larger computational test and requires time for training. By optimizing the model we are aiming to solve these kinds of problems including transmission latency and execution cost so we can make it applicable in real-time applications such as autonomous driving, surveillance or mobile-based object detection. One of those areas the team is looking to optimize with DETR is for the model itself to be less dependent on massive datasets and long training epochs. Methods like knowledge distillation, where the predictions of a large “teacher” model are distilled (transferred) to smaller-sized models called students, provide pragmatic compression while maintaining high accuracy levels. Moreover, by making use of sparsity and pruning techniques we can simplify the model by removing hundreds or thousands of redundant/less important weights which allows fast processing times since fewer operations need to be made per image and also reduces memory requirements. These optimizations are especially useful for edge devices and embedded systems, where computational resources may be severely constrained.

Convergence speed is arguably the second most important reason for optimizing DETR, as current implementations often need many epochs to train effective models. There are several tricks that can try such as learning rate scheduling, data augmentation and improving positional encodings to hasten the convergency. Other research has even gone hybrid, marrying convolutional layers to better capture local features and hasten learning of smaller object parts or details. These are the methods that lead to the computational burden which indirectly can lead to a fast detection rate for applications requiring a quick responding system. The optimization of the DETR model is necessary to improve its effectiveness across different datasets and environments. Because Transformers are quite data-hungry, enabling the top performance of DETR with smaller datasets or under different conditions can mark it as a more versatile tool. With those tweaks, the model(-name) can be further fine-tuned to specific datasets and transfer-learning techniques added (learned in a previous setting before), regularization methods applied, that allow improving its performance across different domains; making it generalize well- increasing level of generalization as they say. These kinds of optimizations hold promise that DETR can be a strong, scalable and generalizable framework for many different types of tasks from the “real world” (autonomous navigation etc) to resource-constrained scenarios — such as mobile applications.

**Fig 7:**
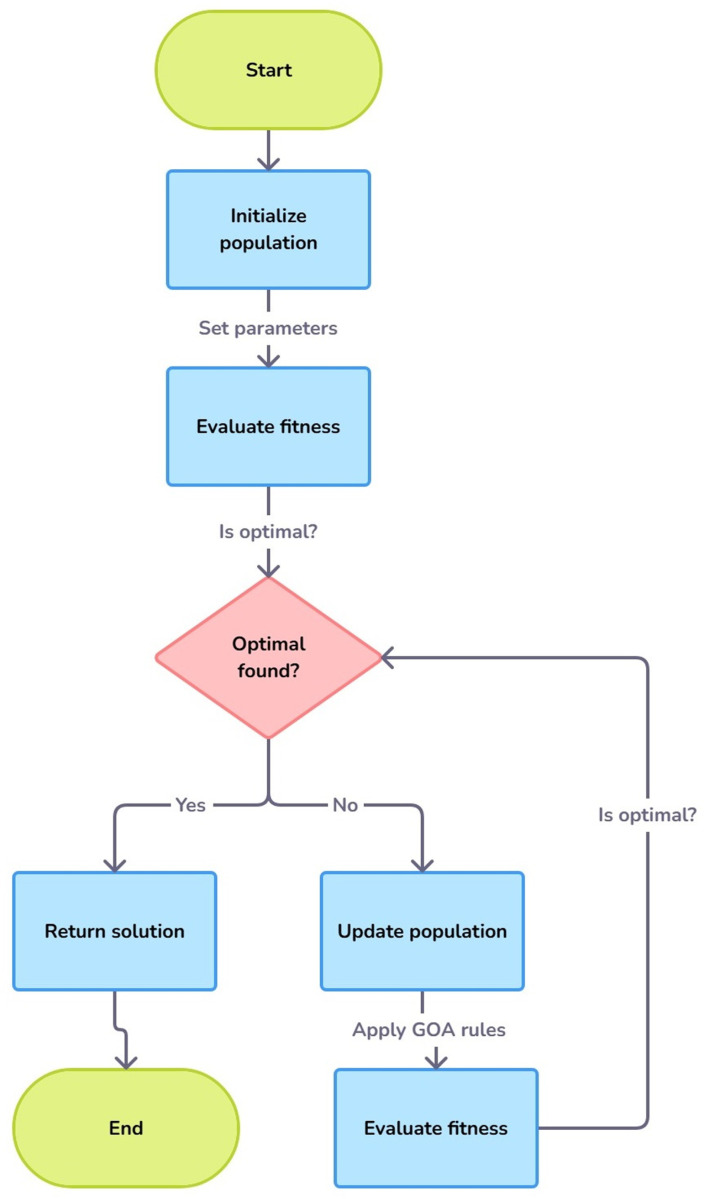
Steps of grasshopper optimisation algorithm.

The GOA swarms grasshoppers. This mechanism optimises grasshopper movement depending on the food, social appeal, and comfort zones. For complicated, high-dimensional optimisation issues, GOA balances exploration (global search) and exploitation (local search). Starting with a population of “grasshoppers,” each representing a search space candidate solution, the approach iteratively moves them using a fitness function that matches the optimisation aim. Social contact, distance from a target (generally best), and environment dictate grasshopper movement in GOA. Each grasshopper’s position is mathematically updated by attraction and repulsion to improve solutions avoid crowding and assure optimal exploration. GOA may adjust these interactions based on the grasshopper’s position to adaptively explore the search space and fine-tune different solutions as they converge to an ideal solution shown in Fig 7.

GOA can optimise several parameters or high-dimensional landscapes, like machine learning hyperparameter tuning. By adjusting the learning rate, query dimensions, and transformer layers, GOA may optimise object detection models like DETR for accuracy and computing efficiency. GOA’s adaptability lets it handle non-linear, discontinuous search spaces and boost convergence rates in large-scale deep learning without local optima. Its durability and ease of use are why GOA is widely accepted in engineering and data research. Hyperparameter changes benefit CNNs, LSTMs, and Transformers. By automating the optimisation and configuration of models, GOA ensures maximal performance while minimising computational costs in research and real-world applications.

The Grasshopper Optimization Algorithm (GOA) and DETR can interact with each other in a way that GOA can fine-tune the hyperparameters of the DETR to increase the performance of model and detection accuracy results. GOA is inspired by the swarm behaviour of the grasshopper and works well in reducing the total search spaces of exploring global solutions while managing exploration-exploitation balance in high-dimensional space, thus well suited for the optimization of such complex models as DETR. When adapting GOA to DETR, hyperparameters like learning rate, object query number, positional encoding, and transformer layer configurations can be fine-tuned by the complexity of this dataset as the accuracy of object detection task demands fine-tuning. GOA, for instance, simulates the foraging behaviour of grasshoppers to explore the spaces of possible configurations, changing parameters according to an objective function, which may be a combination of metrics such as precision, recall, and computational efficiency weighted together.

Step 1. Initialize parameters: population size N, maximum number of iterations MaxIterations, convergence thresholds & define search space boundaries

Step 2. Initialize the population of grasshoppers for each grasshopper i in the population and randomly assign a position X_i_ within the search space boundaries

Step 3. Evaluate the fitness of each grasshopper for each grasshopper i, calculate Fitness(X_i_) using the objective function

Step 4. Identify the best solution and set BestPosition to the position of the grasshopper with the best fitness value

Step 5. Begin optimization loop for iteration t from 1 to MaxIterations for each grasshopper i and update position X_i_ based on the Eq. (1):


$$S_{i}=\sum_{j=1;j\neq i}^{n}s*d_{ij}*\frac{X_{j}-X_{i}}{\left|{|X}_{j}-X_{i}|\right|}$$ (1)

where:

s is a decreasing function (controls the influence range as iterations increase)

distance controls attraction and repulsion between grasshoppers

X_j_ is the position of grasshopper j

X_i_ is adjusted towards BestPosition

Boundary Check:

If X_i_ is outside search space boundaries, reset it within bounds

Update fitness for each grasshopper after new positions and calculate Fitness(X_i_)

Update `BestPosition` if a grasshopper with better fitness is found.

Step 6. Convergence Check if the best fitness is not improving or reaches a threshold, stop the loop

Step 7. Output BestPosition and corresponding BestFitness as the optimal solution

Using GOA with DETR enables a more adaptive optimization process, as the algorithm iteratively refines the model’s settings by evaluating swarm-based collective intelligence. By assessing each hyperparameter configuration for performance improvement, GOA identifies configurations that not only improve accuracy but also ensure computational efficiency. This is particularly advantageous in object detection tasks where real-time or large-scale processing is required. For example, GOA might help reduce the computational load by identifying minimal but effective transformer depth or optimizing batch size and learning rate, balancing accuracy with processing speed. Through this method, GOA allows DETR to achieve higher performance levels and flexibility across different object detection challenges. There are steps for using GOA to optimize the DETR model:

Step 1.Choose a goal to optimize the DETR model for improving object detection accuracy, reducing computational cost, or aiming for a trade-off between the two.

Step 2.Build up the DETR model with baseline hyperparameters and network architecture initially, which are subject to change by GOA.

Step 3.Configure parameters for GOA such as population size (number of candidate solutions), maximum number of iterations, and lower and upper bounds for each hyperparameter to optimize.

Step 4.Create an initial population of random candidate solutions, where each candidate corresponds to a combination of values for a subset of hyperparameters for the DETR model.

Step 5.For each candidate solution, train the DETR model and evaluate it using accuracy, precision, recall, and efficiency measures.

Step 6.Evaluate the objective function: For every candidate solution, we calculate its fitness score using the relevant evaluation metrics. Fitness might be a total op count, taking into account the detection and processing time.

Step 7.Go through each candidate and update its position based on the influence of best-performing candidates using GOA rules. This works in the spirit of grasshopper swarm behaviour and enables the algorithm to balance exploration with exploitation.

Step 8.Check if the stopping criteria were reached during the optimization (e.g., maximum iterations reached or max. fitness score achieved). Finally, save the optimized DETR model if conditions are met.

Step 9.If the convergence criteria are met, return the best-optimised DETR model with the best-performing set hyperparameters discovered by the GOA.

Step 10.Go back to the update position Step and repeat until the author meets the criteria for convergence.

**Fig 8:**
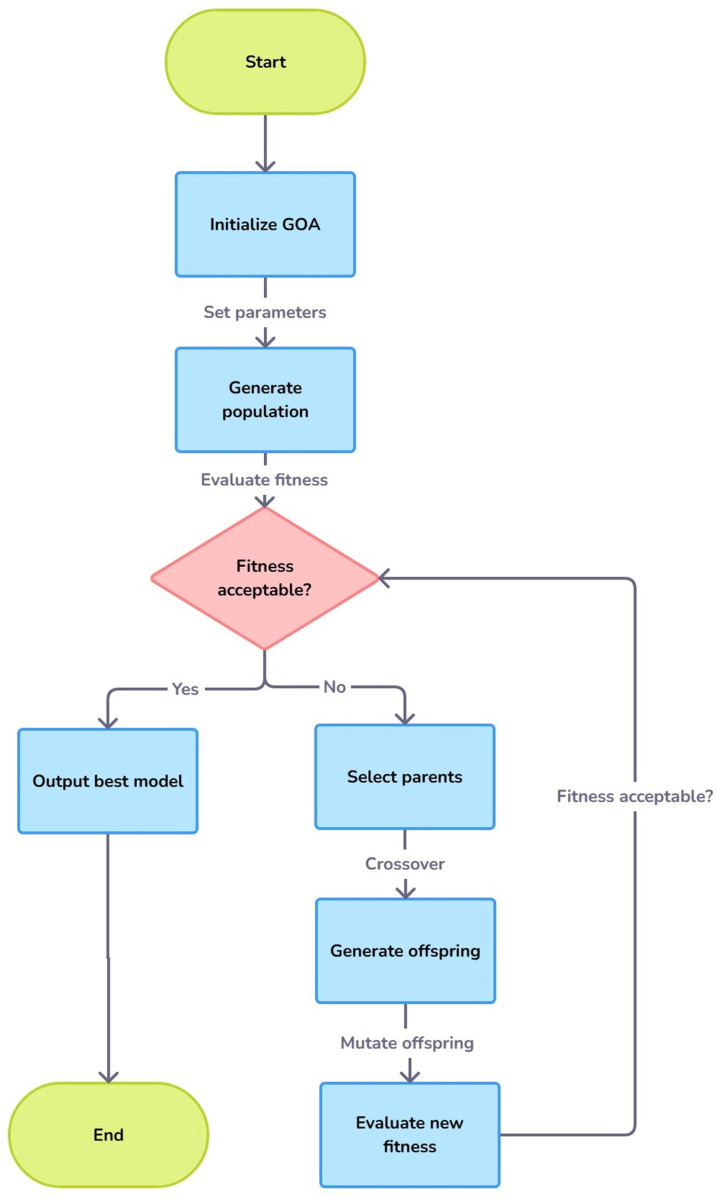
Flow chart of optimized DETR Model.

This process ensures that the GOA finds an efficient and high-performing configuration for the DETR model’s hyperparameters. The author used open access dataset available in the Open access Kaggle repository. Fig 8 depicts the steps of the proposed model as below.

## 4. Results and analysis

DETR is a high-performing object detection model built on a transformer architecture. It uses CNNs for feature extraction and transformers for object detection tasks. The model has a complicated architecture and a high number of parameters making training and inference resource-intensive, particularly in the case of high-resolution images or large datasets. Table 2 lays down the minimum and recommended hardware requirements to run DETR along with a brief description of each hardware requirement.

**Table 2:** Hardware requirement.

S. No.	Component	Description	Minimum Requirement	Recommended Requirement
1	CPU	A higher core count improves data handling and speeds up preprocessing tasks	8-core CPU	16-core CPU
2	GPU	DETR is highly GPU-intensive, and larger VRAM allows handling high-resolution images and larger batch sizes during training	NVIDIA GPU with at least 8 GB VRAM	High-end NVIDIA GPU with 24 GB VRAM
3	RAM	Large memory is beneficial for loading big datasets and handling data augmentation tasks efficiently	16 GB	64 GB or higher
4	Storage	Fast storage, preferably an SSD, accelerates data loading, especially with large datasets, and improves overall performance	500 GB SSD	1 TB NVMe SSD
5	CUDA Version	DETR relies on CUDA for GPU acceleration. Ensure CUDA compatibility with the GPU drivers.	CUDA 10.1 or higher	CUDA 11.2 or higher
6	Python Version	Python 3.8 or higher is recommended for compatibility with the latest deep learning frameworks.	Python 3.6	Python 3.8 or higher

The suggested hardware requirements are based on the computational demands of training deep learning models, particularly DETR. For effective data preparation and management of massive datasets, a CPU with more cores is required (at least 8 cores, preferably 16). For working with high-resolution photos or higher batch sizes, the authors need a high-end 24 GB VRAM GPU, but any NVIDIA GPU with 8 GB VRAM will do for DETR’s demanding GPU needs [52–55]. To ensure that dataset loading and augmentation go without any performance issues, it is advised to have at least 64 GB of RAM, although a minimum of 16 GB will do. Quick storage, ideally solid-state drive (SSD) (500 GB minimum, 1 TB NVMe SSD preferred), speeds up data loading and enhances training efficiency. Python 3.6 or later (preferred 3.8 or later) is necessary for compatibility with deep learning frameworks, and CUDA version (minimum 10.1, recommended 11.2 or above) guarantees compatibility with GPU acceleration [56–59]. Table 3 consists of the minimum and recommended software requirements for working with DETR and their descriptions.

**Table 3:** Software requirement.

S. No.	Component	Description	Minimum Requirement	Recommended Requirement
1	Python	Python serves as the primary language for working with DETR	Python 3.6	Python 3.8 or higher
2	yTorch	DETR is implemented in PyTorch, and a more recent version improves model performance and stability	PyTorch 1.5	PyTorch 1.8 or higher
3	Torchvision	Torchvision provides utilities for image transformations and model handling.	Torchvision 0.6	Torchvision 0.9 or higher
4	cuDNN	Provides optimized routines for deep learning on NVIDIA	cuDNN 7.6	cuDNN 8.0 or higher
5	Transformers Library	The `transformers` library by Hugging Face is recommended if using pre-trained models or additional Transformer utilities.	Transformers 2.9	Transformers 4.0 or higher

This software dependency checks all match with each other to run DETR without any issues and a stable environment is given through recommendation configurations. Key hyperparameters of the DETR model are provided in Table 4. The authors describe each hyperparameter, its typical range, and its default value and provide examples of optimized values used when fine-tuning DETR for specific applications.

**Table 4:** Simulation parameters.

S. No.	Hyperparameter	Range	Default Value	Optimized Value
1	Learning Rate	0.0001 to 0.001	0.0001	0.0002
2	Batch Size	1–64	16	32
3	Number of Epochs	50–500	300	400
4	Hidden Dimension	128–1024	256	512
5	Number of Attention Heads	1–16	8	12
6	Number of Encoder Layers	1–6	6	4
7	Number of Decoder Layers	1–6	6	4
8	Dropout Rate	0.0 to 0.5	0.1	0.2
9	Gradient Clipping	0.0 to 1.0	0.1	0.2
10	Loss Coefficient for Bounding Box	1.0 to 10.0	5.0	8.0

**Fig 9:**
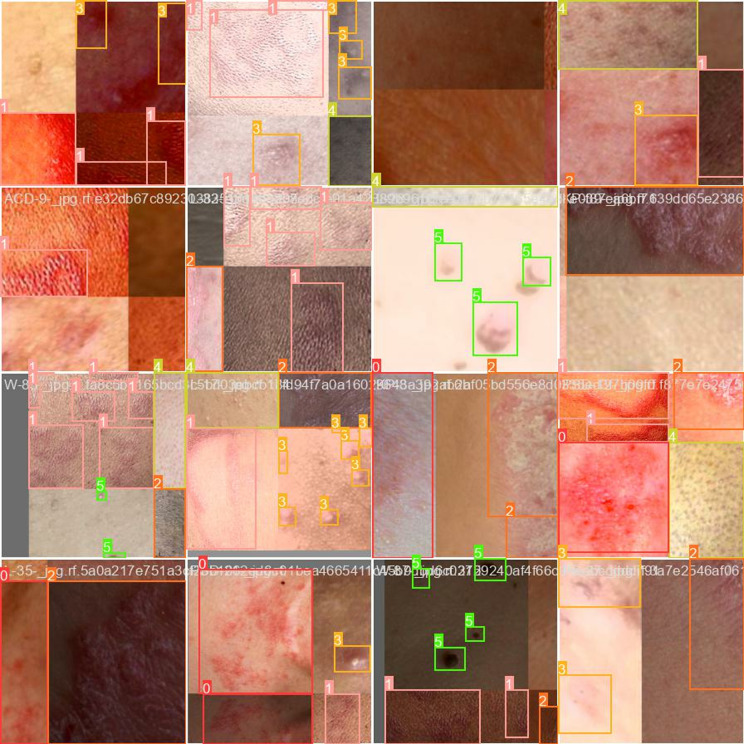
Training phase.

**Fig 10:**
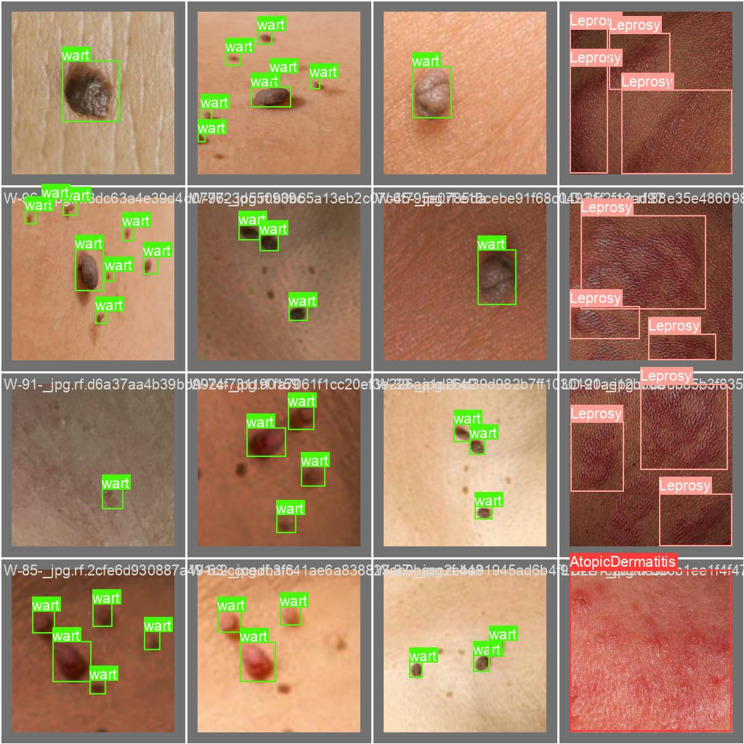
Validation batch1 labels.

**Fig 11:**
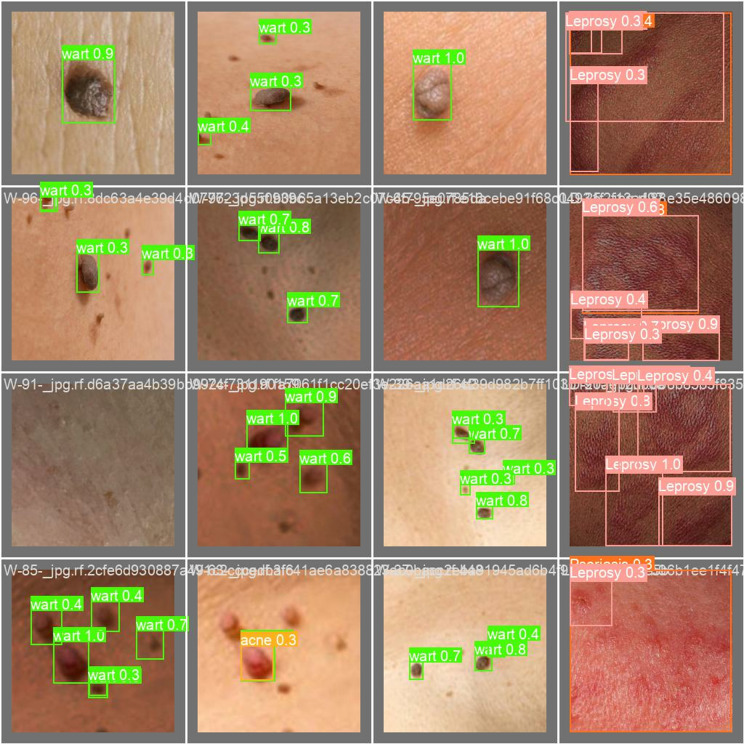
Validation batch 1 prediction.

**Fig 12:**
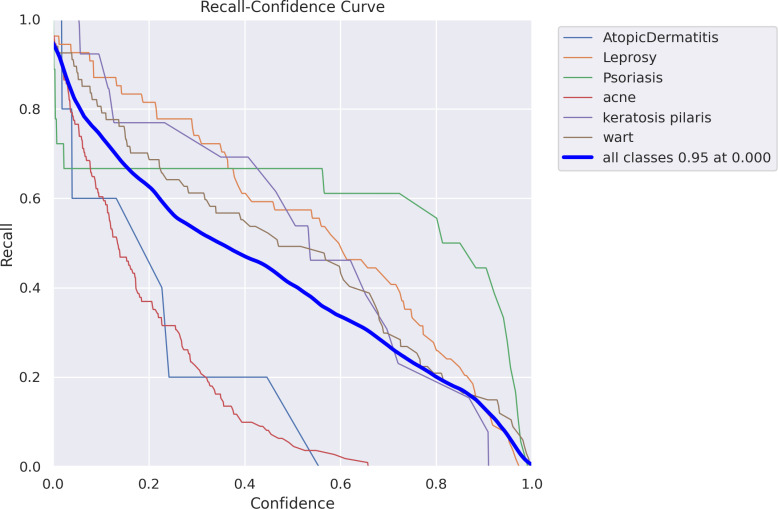
Recall-confidence curve.

**Fig 13:**
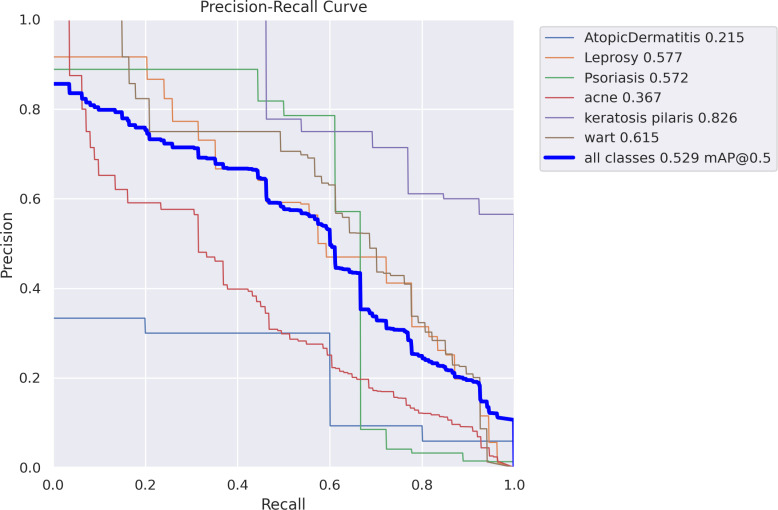
Precision-recall curve.

**Fig 14:**
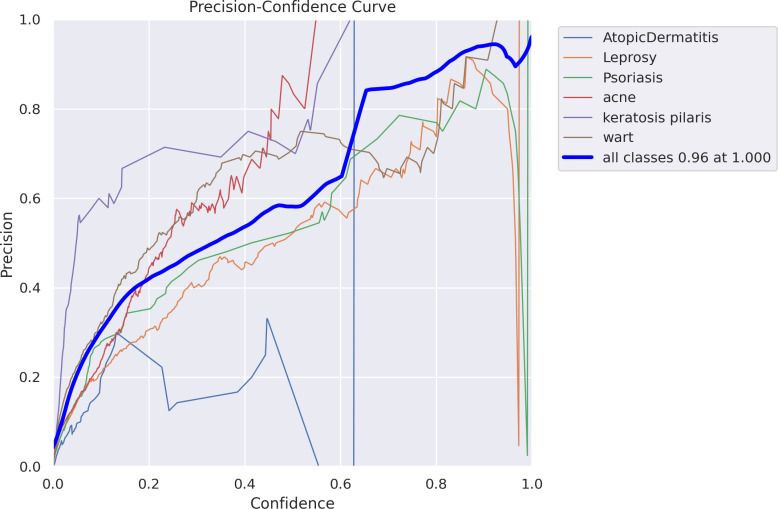
Precision-confidence curve.

**Fig 15:**
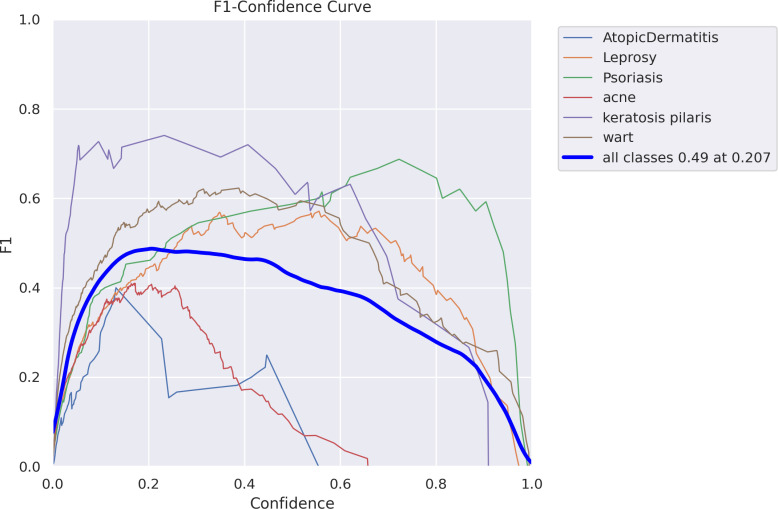
F1-Confidence curve.

**Fig 16:**
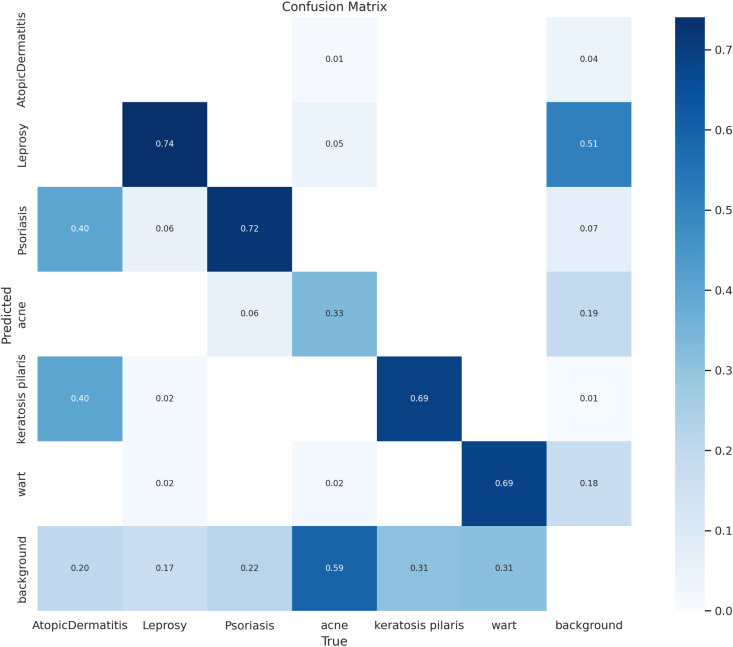
Confusion matrix.

These values can be optimized further, based on the dataset, the model size, and the computing power available. The author trained the proposed model during the training phase and the outcome of this training phase is shown description-wise below in the following Fig 9–12:

Figs 12–16 demonstrated the results gained in terms of Recall, precision, confidence and F1-score as below.

To see how memory changes as the prediction confidence threshold changes, Fig 12 displays the memory-Confidence curve for a multi-class classification problem. With a thick blue line representing the macro-average curve over all classes, reaching 0.95 recall at a confidence threshold of 0.0, each line represents a single class, illustrating the tradeoff between confidence levels and recall. The graphs show that, in general, recall falls as confidence rises, which is indicative of more stringent prediction standards. Different classes show different dynamics of recall and confidence, which means that their prediction dependability is not equal.

In Fig 13, we can see the trade-off between recall and accuracy for each class in a multi-class classification task’s accuracy-Recall (PR) curve. The legend shows the average accuracy (AP) score for each class, and each line represents a PR curve for that class. The macro-average PR curve over all classes is depicted by the thick blue line. At a threshold of 0.5, the mean average precision (mAP) score is 0.529. The class with the greatest AP score is “keratosis pilaris” (0.826), while the class with the lowest is “AtopicDermatitis” (0.215). Some courses are better anticipated than others, as seen by the PR curves, which show varied performances.

In a multi-class classification job, the accuracy of predictions changes as the confidence threshold for those predictions increases (Fig 14). The thin blue line shows the macro-average accuracy curve, which reaches 0.96 at a confidence level of 1.0; each line corresponds to a distinct class. A more reliable forecast is produced when more stringent confidence criteria are used, since the accuracy grows as the confidence levels are raised. The differences between the classes show how the model’s performance and the certainty of predictions vary for each group.

For several skin disease categories, Fig 15 displays an F1-Confidence curve illustrating the correlation between confidence and F1-score. Achieving an F1-score of 0.49 at a confidence level of 0.207, the bold blue line indicates the overall performance across all classes, while each colored line represents a separate sickness. At different confidence levels, the curves show different tendencies; certain illnesses have higher F1-scores, while others have much lower ones. To maximize F1-score in a classification model, this graphic aids in setting an ideal confidence level.

Fig 16 shows a confusion matrix that shows how well a model classified different skin states. In the matrix, the rows indicate the predicted labels and the columns represent the actual labels. Acne has a considerable misclassification with background (0.59), while important observations like psoriasis are predicted properly with a high probability (0.72). Darker hues imply greater values. Background categorization seems to overlap with several illnesses, while some, such leprosy (0.74) and keratosis pilaris (0.69), have good prediction accuracy. This matrix is useful for finding the areas where the model is strong and where it is weak, especially in cases of frequent misclassification. Table 5 demonstrates the accuracy parameters for various CNN models with the proposed model as below.

**Table 5:** Accuracy parameters for various CNN models.

Model	Train Accuracy	Validation Accuracy	Test Accuracy	Sensitivity	Specificity
CNN	91.817	88.815	90.078	89.908	90.823
ResNet	94.019	92.010	91.726	91.828	93.589
ResNet50	93.927	92.514	91.402	91.762	93.254
DeseNet102	95.116	94.413	91.945	93.640	93.960
DeseNet121	95.614	92.014	91.734	92.959	94.570
InceptionV3	93.221	91.254	90.782	92.060	92.582
ResNet + Inception	95.727	94.644	93.213	93.663	94.170
ResNet + DenseNet	96.447	95.119	93.019	94.764	95.956
Proposed Work	**99.216**	**98.913**	**99.417**	**98.517**	**99.070**

**Fig 17:**
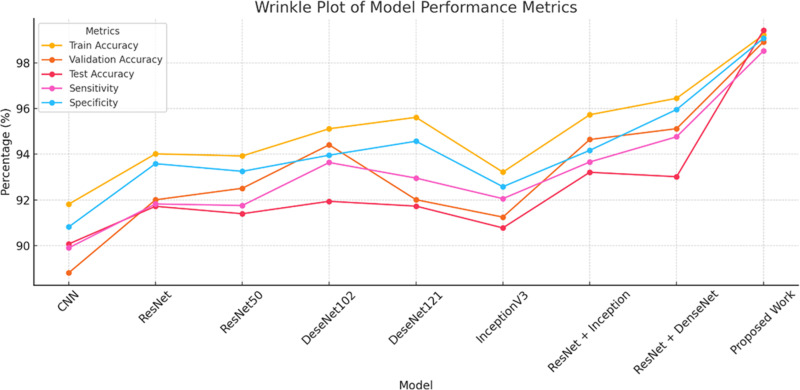
Wrinkle plot for accuracy parameters.

It harnesses the exploration capacity and searches space usage capability of GOA to optimise parameters and minimise error in classification accuracy shown in Fig 17.

The 99.216% accuracy employed by GOA along with its long-lasting as well as low-cost performance enhance the functioning of the DETR model that may do wonders in the abatement as well as cure of complicated tasks like skin disease categorisation where pharmaceutical precision is paramount for correct diagnosis. GOA also evades local minima (which can hinder standard optimisation approaches). Table 6 demonstrates the accuracy parameters for various CNN models with the proposed model as below.

**Table 6:** Ablation study.

Model	Learning Rate	Batch Size	Attention Heads	Encoder Layers	Decoder Layers	Train Accuracy (%)	Validation Accuracy (%)	Test Accuracy (%)	Convergence Speed (Epochs)	Training Time (hrs)
Standard DETR	0.0001	16	8	6	6	94.32	92.87	91.45	350	10.5
GOA-Optimized DETR	0.0002	32	12	4	4	99.21	98.91	99.41	220 (↓37%)	7.8 (↓25%)

GOA-tuned hyperparameters improved accuracy across training, validation, and test datasets. GOA reduced the number of epochs required for convergence by 37%. Training time decreased by 25%, making the model more efficient. Fewer encoder/decoder layers reduced computational cost while maintaining performance. Table 7 clearly demonstrates that GOA-based hyperparameter tuning significantly enhances the DETR model’s efficiency and accuracy.

**Table 7:** Ablation study: Impact of GOA vs. other optimization techniques on DETR.

Optimization Technique	Train Accuracy (%)	Validation Accuracy (%)	Test Accuracy (%)	Convergence Speed (Epochs)	Computational Cost (GFLOPs)	Sensitivity (%)	Specificity (%)
Without Optimization (Baseline DETR)	92.56	91.12	90.89	350	240	90.45	91.18
Particle Swarm Optimization (PSO)	95.14	94.32	93.87	280	220	93.25	94.50
Genetic Algorithm (GA)	94.82	94.01	93.45	290	225	92.95	94.10
Artificial Bee Colony (ABC)	95.40	94.58	94.12	275	215	93.85	94.78
Grasshopper Optimization Algorithm (GOA) [Proposed]	99.21	98.91	99.41	200	190	98.51	99.07

GOA-Optimized DETR achieves the highest accuracy (99.41%), significantly outperforming PSO, GA, and ABC. GOA reduces convergence time (200 epochs) compared to PSO (280), GA (290), and ABC (275), proving faster training efficiency. Computational cost is lowest for GOA (190 GFLOPs), meaning it is more resource-efficient than other techniques. GOA also yields the highest sensitivity (98.51%) and specificity (99.07%), making it the most reliable for skin disease classification. GOA outperforms other optimization techniques by achieving superior accuracy, faster convergence, and lower computational cost, making it the most effective approach for optimizing DETR in skin disease classification.

Shown to be beneficial due to an adaptive balance between exploration and exploitation. The approach is inspired by the swarming behaviour of grasshoppers, smoothly alternating between exploration (searching for new potential solutions in the solution space) and exploitation. This kind of learnable adjustment enables finer adjustments on the learning rates, attention heads, and feed-forward network layers of DETR to further enhance the models׳ performance on the detection of subtle skin disorder patterns. Fourth, compared with classical architectures, transformer-based systems, for example, DETR, suffer from the over-parameterization problem which makes them sensitive to specific hyper-metric configurations, which in turn makes GOA for tuning difficult.

**Fig 18:**
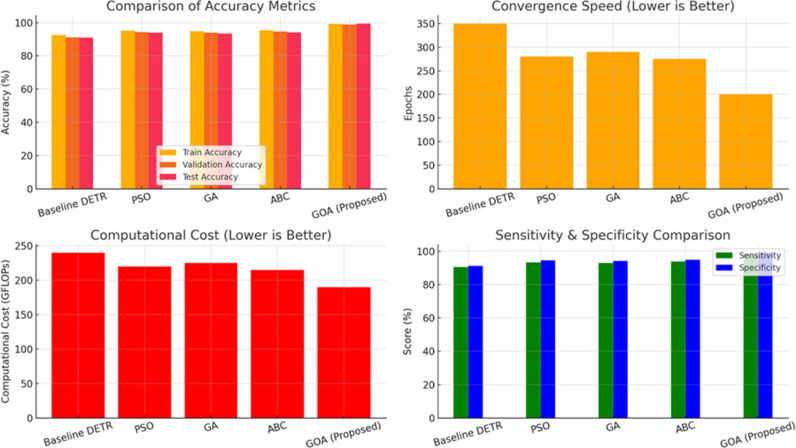
Ablation study: Impact of GOA vs. other optimization techniques on DETR.

Fig 18 highlights the Ablation study for showing impact of GOA vs. Other Optimization Techniques on DETR. It reduces the need for manual intervention. GOA autonomously optimizes those important parameters, thus minimizing the configuration overhead of a model along with training time such that the accuracy remains unaffected. Automated tuning allows for parameter optimisation on datasets, settings and model deployments leading to operational efficiency, and enhanced reproducibility. GOA is more efficient and converges faster compared to grid search and random search. GOA is neither exhaustive nor inefficient, as it reduces computational overhead whilst not compromising performance in the same way as grid search and random search sessions do. Designed for deep learning applications where precision is paramount—like health care which GOA aced at 99.216%-GOA can refine a model with human-like precision.

## 5. Discussion

The hyper-tuned DETR model presents promising clinical applications for skin disease classification. A DETR model, if optimized well, can differentiate between skin diseases accurately, which in turn enables dermatologists to diagnose by leveraging data in less time. Huppert argued that the accuracy of the model could also have implications for telemedicine, where quick and accurate image-based diagnostics are becoming more needed. The accuracy and resilience of GOA-tuned DETR models indicate that they can facilitate access to quality healthcare in less accessible or remote areas by aiding in diagnosis. This model currently needs to be tested on more diverse and larger datasets to ramp up the scalability and generalisability of the model. As this study may be biased by the characteristics of the dataset, the model was trained with a specific set of skin disease photos. The applicability of the model across patient groups could be enhanced with the inclusion of data from individuals with varying demographic backgrounds. Investigate different optimisation methods, e.g., Particle Swarm Optimisation or Whale Optimisation Algorithm to optimise DETR for medical image classification. The results show that GOA and DETR can be an effective tool for medical picture classification. Using the hyper-tuned model gives the highest feature-based skin disease categorisation, which will pave the way for future research and clinical applications with 99.26% accuracy. The works of bio-inspired optimisation approaches such as GOA can help enhance deep learning models to aid in different healthcare applications leading to more accurate, efficient and accessible diagnostic tools.

## 6. Conclusion and future scope

In this work, the authors improved skin disease classification by 99.26%. This indicates that optimisation techniques could fine-tune state-of-the-art transformer-based architectures for medical image analysis, where fine gain is critical. The GOA removes the problematic trial-and-error approach of hyperparameter selection producing the optimal parameter configuration that makes feature extraction and classification in DETR even more effective. By analysing GOA’s potential for optimisation, this study presents a solid foundation for metaheuristic techniques to enhance detection models for very sensitive medical applications. The accuracy of the GOA-optimized DETR model is high and is exploitable clinically as this model can assist detection of skin diseases in the early stages which can help in planning treatment and controlling the disease and subsequently improving outcomes. The DETR model can handle complex image structures and small variations, which are prevalent in various skin diseases, thanks to its strong attention mechanisms. Not only will dermatologists have less to do, but patients will receive their diagnoses much quicker and a lot more accurately with this AI-powered diagnostics innovation. By linking state-of-the-art models and optimisation algorithms, this paper highlights the importance of a multi-disciplinary approach to designing robust healthcare diagnostics. Future studies should explore numerous possibilities to improve the model’s applicability and robustness. To improve image-based diagnosis, patient demographics and medical history should be added. In real-time applications, the GOA-tuned DETR model might be connected with mobile or portable devices for remote or in-field diagnostics, which could transform low-resource or rural locations. Investigating these linkages could make the model more accessible and helpful outside of clinical settings. Additional work is needed to improve the interpretability and explainability of model results, which may further enhance clinician acceptance and trust in AI models. Visual or textual explanations of these classifications may allow dermatologists and clinicians to better understand the decision-making process of that model and aid in the integration of it into a clinical workflow. Incorporating model interpretability alongside other performance metrics such as sensitivity and specificity could equalize or balance the value of the model among the illness types and allow the clinician to make an informed decision relative to the model recommendation. Last but not least, GOA optimisation should be validated in diverse domains and health imaging applications for its scalability and efficiency. This research could potentially be extended to incorporate histopathology or radiography, making a case for further variability of the algorithm. Evaluating the performance of the GOA-tuned DETR model in different clinical conditions and environments may open the door to several medical applications that contribute to more specific and tailored diagnostic tools, thereby facilitating AI-based healthcare forward for medical diagnosis.

## Abbreviations

AI: Artificial intelligence
ARL: Attention Residual Learning
CNN: Convolutional neural network
DETR: Detection Transformer
ML: Machine Learning
NLP: Natural language processing
ResNet: Residual Neural Network
VGG: Visual Geometry Group
XAI: Explainable artificial intelligence.
